# Association between ambient temperature and non-accidental mortality in Guiyang, China: A time-series analysis (2013-2023)

**DOI:** 10.1371/journal.pone.0319863

**Published:** 2025-04-01

**Authors:** Xuanhao Chen, Minmin Su, Minlan Yuan, Zihai Jian, Dan Yang, Hua Guo, Jianhua Zhang

**Affiliations:** Guizhou Center For Disease Control And Prevention, Guizhou, China; University of Rajshahi, BANGLADESH

## Abstract

**Background:**

As climate change intensifies, ambient temperatures have become a global concern, leading to an increasing number of studies examining the impact of temperature on human health. Extreme weather events, including heatwaves and cold spells, are becoming more frequent and severe. Numerous studies have highlighted the positive correlation between non-optimal ambient temperatures and mortality. Understanding these impacts is crucial for developing targeted public health interventions and accurately predicting the future health burden associated with climate variability. This study aims to estimate the relative risks and mortality burden associated with temperature extremes over the past decade, focusing on the contributions of both heat and cold, as well as mild and extreme temperatures, and identifying vulnerable populations. By doing so, filling a regional research gap in Guiyang.

**Methods:**

We collected the daily weather and mortality data from 2013 to 2023. Descriptive analysis was conducted to characterize overall weather patterns and mortality trends during the study period. A quasi-Poisson regression with a distributed lag non-linear model (DLNM), incorporating a 21-day lag and controlling for trends, air pollutants, and the day of the week, was applied to estimate the cumulative relative risks of non-accidental mortality due to non-optimal and extreme temperatures. We calculated attributable fractions and attributable numbers for heat and cold (defined as temperatures above and below the daily mean temperature), mild temperatures (defined using cutoffs at the minimum mortality temperature, with mild heat ranging from the minimum mortality temperature to the 97.5th temperature percentile and mild cold ranging from the 2.5th temperature percentile to the minimum mortality temperature) and extreme temperatures (defined as temperatures below the 2.5th temperature percentile for extreme cold and above the 97.5th temperature percentile for extreme heat).

**Results:**

A total of 140,099 non-accidental deaths were included in the study.Temperature and mortality showed U-shaped associations, except for 0-64 years age group. For extreme low temperatures, the effects appeared in lag 2 to 4 days and lasted for approximately 18 days, peaking on lag day 5, yielding a cumulative relative risks (RRs) of 1.24% (95% CI 1.14% to 1.36%) for non-accidental mortality. For extreme high temperatures, the strongest effect was observed on the same day, with an RR of 1.18%(95% CI 1.03% to 1.35%). The attributable fraction of non-accidental mortality associated with non-optimal temperatures was 9.21% (95% eCI: 5.32% to 12.15%). The mortality burden from heat and cold was 5.55% (95% eCI: 2.04% to 8.59%) and 3.67% (95% eCI: 1.45% to 5.80%), respectively. Mild heat was responsible for the majority of the mortality burden.

**Conclusion:**

Extreme low temperatures had higher cumulative relative risk and a prolonged effect compared to extreme high temperatures. The attributable fraction associated with non-optimal temperatures was highest for respiratory-related deaths. Mild heat was responsible for the majority of the mortality burden. Additionally, males and the individuals aged 65 years and above were particularly vulnerable populations.

## Introduction

Climate change represents a critical global challenge, primarily driven by anthropogenic activities such as fossil fuel combustion, and carbon dioxide emissions [1,2]. The resulting increase in greenhouse gases has led to rising global temperatures, disrupted weather patterns, and heightened frequency of extreme weather events. Non-optimal temperatures are recognized as a significant risk factor, warranting extensive research to better understand its health-related consequences. This information is essential for the development of targeted public health strategies and the provision of accurate climate change impact forecasts.

The relationship between temperature and mortality is a critical area of public health research, highlighting the profound influence of environmental conditions on human health and survival. As global temperatures continue to rise due to climate change, understanding this relationship becomes increasingly important. Extreme temperatures, both hot and cold, can lead to a variety of health issues, including heat strokes and exacerbations of chronic illnesses, particularly affecting the cardiovascular and respiratory systems [3–5]. Vulnerable populations, such as the elderly, children, and those with preexisting health conditions, are especially at risk [6–8].

Given the regional variability in climate conditions and population health status, it is important to conduct localized studies to better understand these specific contexts. Such research is essential for developing public health strategies that address the unique needs and challenges of each region, ensuring more effective and relevant interventions.

By addressing this regional research gap in Guiyang, we aim to explore the relative risks and mortality burden associated with non-optimal ambient temperatures over the period from 2013 to 2023, as well as the relative contributions from heat and cold, and from mild and extreme temperatures. Additionally, we seek to identify vulnerable populations by stratifying the data according to cause-specific diseases, age, and gender.

## Methods

### Study area and data sources

Guiyang, the capital of Guizhou Province in southwest China (106°07′ E to 107°17′ E, 26°11′ N to 27°22′ N), spans a total area of 8,043km^2^. Located on the eastern side of Yunnan-Guizhou Plateau, Guiyang experiences frequent rainfall and high humidity throughout the year due to its position under a quasi-stationary front. Air pollution in Guiyang is relatively low, attributed to its minimal industrial activity and high forest cover. The geographical location of Guiyang within China further influences its climate and environmental characteristics.

Individual death records were retrieved from the Disease Surveillance Points System, administered by the Chinese Center for Disease Control and Prevention, covering the period from January 1,2013, to December 31,2023.The data included a range of causes of death based on the primary diagnosis coded by ICD-10 (international classification of diseases, 10th revision), including non-accidental causes (A00-R99), respiratory cases (J00-J99),cardiovascular cases (I00-I99). We excluded the data from December 18, 2022, to January 28, 2023, due to the COVID-19 outbreak in Guiyang, which disrupted mortality patterns and caused an abnormal increase in deaths, potentially introducing significant bias into the analysis. The data were also stratified into three subgroups: cause-specific mortality (overall,respiratory and cardiovascular), age (0-64years, ≥65 years), and gender (male and female). Daily temperature and relative humidity data from 2013 to 2023 were obtained from the Guiyang Meteorological Bureau.

To adjust for confounding bias due to the effects of air pollutants, daily concentrations of SO_2_ and PM_2.5_ (as indicators of air pollution) from all monitoring stations in Guiyang were obtained from the Guiyang Bureau of Ecology and Environment. The daily pollutants concentration was calculated as the arithmetic mean across all monitoring stations, consistent with standard time-series studies. Missing value, which accounted for less than 1% at each station, were excluded from the analysis.

The dataset does not contain any information that can identify individuals, and this research poses no harm to human health nor involves sensitive personal information. Therefore, ethical approval is not required.

### Statistical analyses

Due to the nonlinear and lag effects, the distributed lag nonlinear model (DLNM) was employed to analyze the relationship between temperature on non-accidental mortality. The primary model utilized a natural cubic spline for the calendar day, with 7 degrees of freedom per year to account for long-term trends and seasonal variations. It also included natural cubic splines for current-day pollutants and humidity, with 6 and 3 degrees of freedom, respectively, as well as a categorical variable for the day of the week. A cross-basis function for daily temperature was incorporated using the DLNM, featuring a natural cubic spline with three internal knots at the 20th, 50th, and 70th percentiles of the temperature distribution. The lag-response curve employed a natural cubic B-spline with an intercept and three internal knots spaced equally on a log scale, with a maximum lag of 21 days, consistent with most studies. [9,10] The DLNM model structure is as follows:


$$\text{log}[E(Yt)]=\alpha +\text{cb}(Temp_{t})+ns(\text{rhum},\text{3})+ns\left(\text{time},\text{7*10}\right)+ns({\text{SO}}_{\text{2}},6)+ns({\text{PM}}_{\text{2.5}},6)+DOW$$

where t denotes the observation day, *Y*_*t*_ represents the observed daily mortality on calendar day *t*, and α is the intercept. The variable *cb(Temp*_*t*_*)* denotes the transformed cross-basis function produced by the DLNM, combining exposure-response and lag-response associations [11]. *ns()* is the natural cubic spline function.*rhum* is relative humidity.*time* is the variable of the temporal trend. *SO*_*2*_ and *PM*_*2.5*_ denote the concentrations of sulfur dioxide and particulate matter on day *t*. The overall cumulative relative risk (RR) and 95% confidence interval (CI) were calculated.

To estimate the mortality burden from non-accidental deaths, we calculated the total attributable fractions (AFs) and attributable numbers (ANs) [12]. The temperature effects were divided into exposure to cold and heat by summing the subsets corresponding to days with temperatures below and above the minimum mortality temperatures (MMTs). Extreme cold and heat were defined as temperatures below the 2.5th percentile and above the 97.5th percentile of the temperature distribution. To estimate the empirical confidence intervals (eCIs) for the attributable fraction and number, Monte Carlo simulations were conducted, assuming a multivariate normal distribution of the best linear unbiased predictions of the estimated coefficients.


$$AF=\sum_{i=0}^{L}\frac{(RRi\text{-1)}}{RRi},\;AN=N*AF$$

where *N* is the annual total counts of deaths, i is the lag day, *L* is the maximum lag day. The R software (version 4.3.3) with the DLNM package by A. Gasparrini.

### Sensitivity analysis

Sensitivity analyses were conducted to evaluate the stability of the estimates. We adjusted the lag days to 7,14, and 21 to determine the appropriate maximum lag. Covariates were assigned varying degrees of freedom to assess the robustness of our estimates: 5 to 8 for air pollutants and 3 to 6 for relative humidity.

## Results

### Descriptive Statistics

Table 1 and Table 2 provide the descriptive statistics for non-accidental mortality and weather conditions in Guiyang from 2013 to 2023. During the study period, we analyzed a total of 140,099 non-accidental deaths, including 83,272 males and 56,827 females. Of these, 102,639 were individuals aged 65 or above, and 37,460 were aged 0-64 years. Deaths due to respiratory diseases and cardiovascular diseases totaled 21,028 and 59,830, respectively. The daily mean temperature ranged from -4.4°C to 28.2°C, while the mean humidity was 80.27%, with a range from 32.00% to 100.00%.

**Table 1:** Distributions of non-accidental mortality in Guiyang, 2013-2023.

Variable	Total	Mean ± SD	Min	P25	P50	P75	Max
Non-accidental deaths	140,099	32.3±16.2	1.0	21.0	37.0	48.0	100.0
Respiratory deaths	21,028	5.3±3.3	0.0	3.0	5.0	7.0	26.0
Cardiovascular deaths	59,830	15.1±7.7	0.0	9.0	15.0	20.5	45.0
Male	83,272	21.0±9.9	0.0	13.0	21.0	28.0	58.0
Female	56,827	14.3±7.5	0.0	8.0	14.0	20.0	42.0
Age 0-64 years	37,460	9.4±4.8	0.0	6.0	9.0	13.0	28.0
Age ≥ 65 years	102,639	25.8±12.7	0.0	15.0	26.0	36.0	73.0

**Table 2:** Descriptive statistics for weather and air pollutants, 2013-2023.

Variable	Mean ± SD	Min	P25	P50	P75	Max
Temperature (°C)	15.3±7.4	-4.4	9.4	16.4	21.8	28.2
Humidity (%)	80.3±11.8	32.0	72.7	81.0	89.7	100.0
SO_2_ (μg/m^3^)	13.8±14.4	3.0	6.0	8.9	15.4	159.0
PM_2.5_ (μg/m^3^)	32.4±21.0	4.8	17.1	27.6	41.6	167.8

### Temperature-mortality relationship

**Fig 1:**
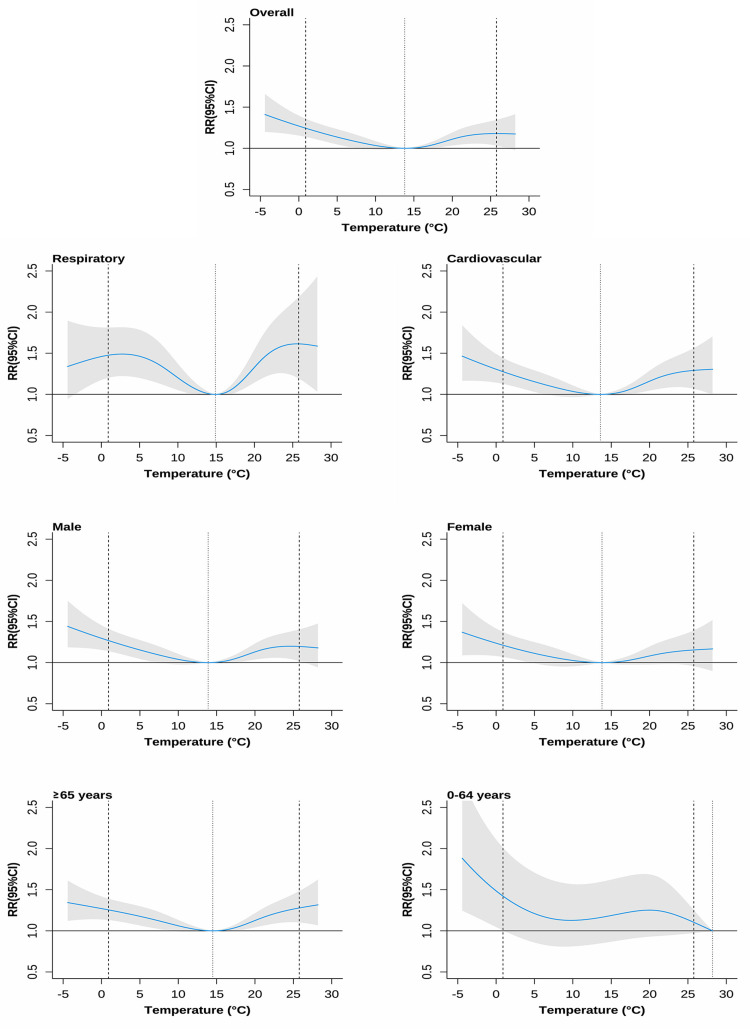
Cumulative exposure-response curves and the relative risks for daily temperature effects on non-accidental mortality over lag days 0-21,2013-2023. The dotted lines are the median of the daily mean temperature, the dashed lines are the 2.5th and 97.5th percentiles of the distribution of daily mean temperature. Shaded areas mean 95% confidence intervals.

The overall effects of temperature on mortality over a 0-21 day period are illustrated in Fig 1. Generally, the curves displayed a U-shape, indicating an increased mortality risk associated with non-optimal temperatures. Mortality risk was higher at lower temperatures and increased as the temperature decreased, with the exception for individuals aged 0-64 years.

**Fig 2:**
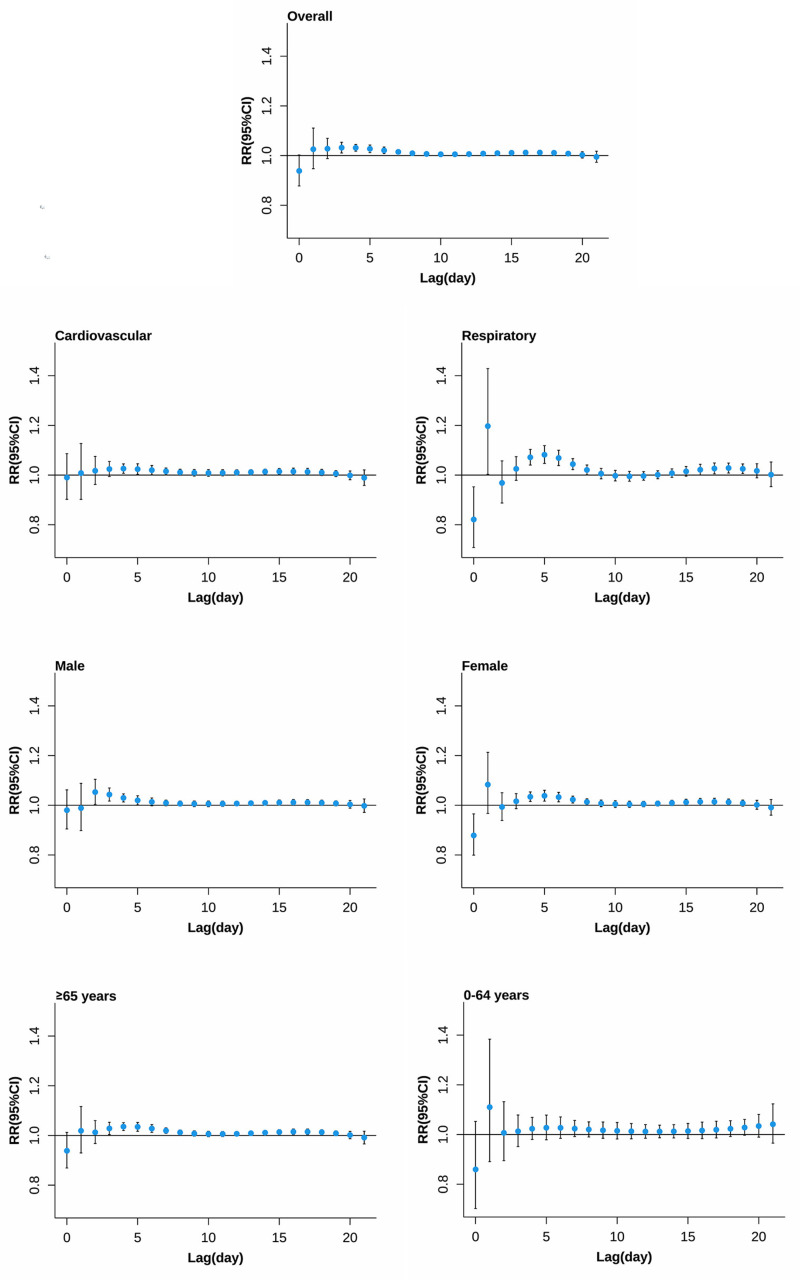
The lag structures in effects of extreme low temperature on non-accidental mortality. Effects were defined as the risks at 0.9°C (the 2.5th percentile of daily mean temperature distributions) compared with the estimated minimum mortality temperature.

**Fig 3:**
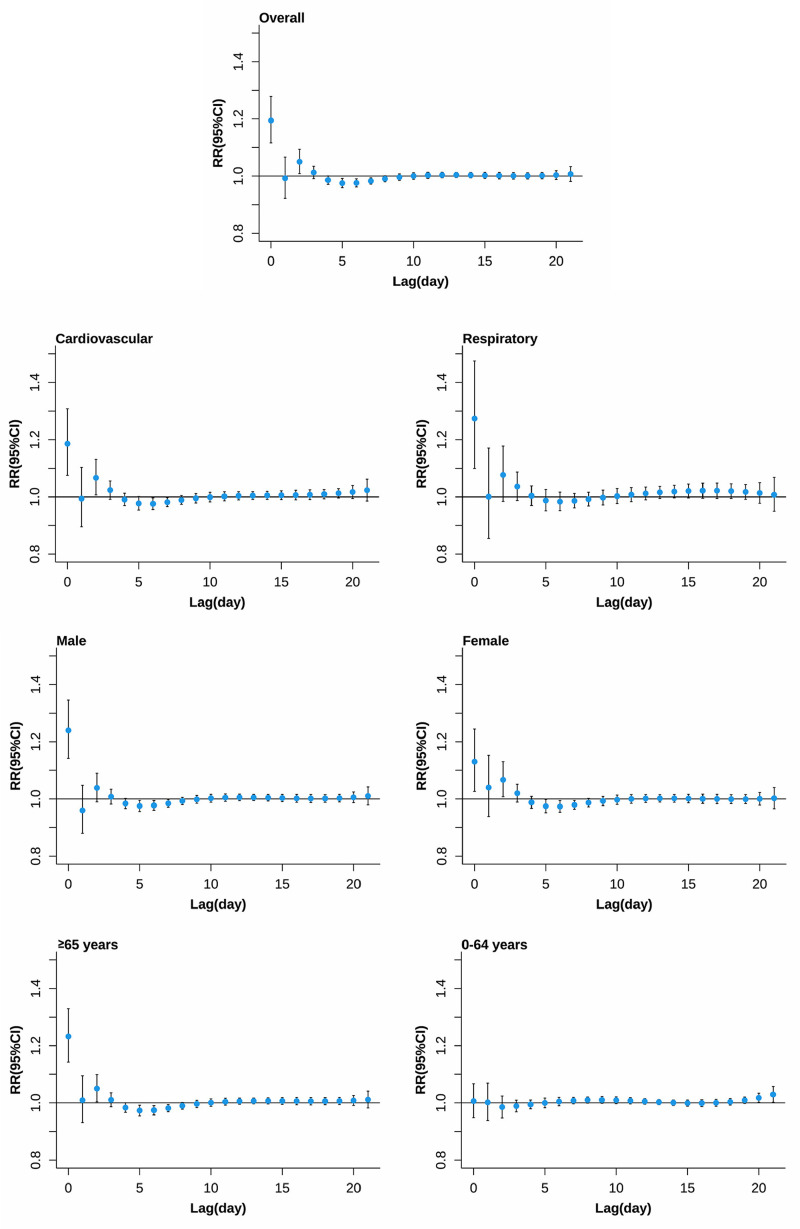
The lag structures in effects of extreme high temperature on non-accidental mortality. Effects were defined as the risks at 25.8°C (the 97.5th percentile of daily mean temperature distributions) compared with the estimated minimum mortality temperature.

Fig 2 and Fig 3 highlight the temporal structure of mortality risk associated with extreme temperatures. For extreme low temperature (0.9°C, 2.5th percentile of daily mean temperature), the risk of all-cause mortality, except for the 0-64 years age group, generally occurred on lag days 2 to 4, peaked on lag day 5, and then gradually decreased, persisting up to lag day 18. For extreme high temperatures (25.8°C, 97.5th percentile of daily mean temperature), except for the 0-64 years age group, the highest mortality risks were observed on the day of exposure, followed by significant mortality displacement on lag days 4 to 7 for almost all causes of death.

Table 3 summarizes the minimum mortality temperatures (MMTs) and the relative risks (RRs) associated with extreme low and high temperatures across different subgroups. The MMTs ranged from 13.6°C to 28.2°C, showing consistency across all subgroups except for the 0-64 years age group. For extreme low temperatures, the relative risks of non-accidental mortality, cardiovascular diseases, respiratory diseases, male, females, those aged ≥ 65 years, and the 0-64 years group were 1.24% (95% CI:1.14% to 1.36%), 1.28% (95% CI:1.13% to 1.44%), 1.48% (95% CI:1.20% to 1.81%), 1.27% (95% CI:1.14% to 1.41%), 1.21% (95% CI:1.07% to 1.37%), 1.25% (95% CI:1.13% to 1.39%) and 1.42% (95% CI:1.01% to 2.01%), respectively. These results indicate that extreme low temperatures are associated with a significantly increased risk of mortality across all subgroups, with the respiratory diseases subgroup and the 0-64 years group showing particularly high relative risks. For extreme high temperature, the relative risks of non-accidental mortality, cardiovascular diseases, respiratory diseases,males and those aged ≥65 years were 1.18% (95% CI:1.03% to 1.35%), 1.29% (95% CI:1.07% to 1.56%), 1.61% (95% CI:1.19% to 2.18%), 1.20% (95% CI:1.02% to 1.40%) and 1.28% (95% CI:1.10% to 1.48%), but no statistically significant results were observed for females and the 0-64 years age group.

**Table 3:** Minimum mortality temperatures (MMTs) and relative risks (RRs) associated with extreme low and high temperatures with 95% empirical confidence interval (95% eCI).

groups	The minimum mortality temperature (°C)	Extreme low temperature (%)	Extreme high temperature (%)
Overall	13.8	1.24 (1.14 to 1.36)	1.18 (1.03 to 1.35)
Respiratory disease	14.9	1.48 (1.20 to 1.81)	1.61 (1.19 to 2.18)
Cardiovascular disease	13.6	1.28 (1.13 to 1.44)	1.29 (1.07 to 1.56)
male	13.9	1.27 (1.14 to 1.41)	1.20 (1.02 to 1.40)
female	13.8	1.21 (1.07 to 1.37)	1.15 (0.96 to 1.39)
≥65 years	14.5	1.25 (1.13 to 1.39)	1.28 (1.10 to 1.48)
0-64 years	28.2	1.42 (1.01 to 2.01)	1.10 (0.97 to 1.25)

### Attributable fractions and attributable numbers

As shown in Table 4, the attributable fraction of non-accidental mortality associated with non-optimal temperature was 9.21% (95% eCI: 5.32% to 12.15%). Subgroup analysis revealed the highest attributable fraction in respiratory diseases 23.80% (95% eCI: 15.60% to 30.36%), followed by cardiovascular diseases 11.99% (95% eCI: 6.58% to 17.15%). Gender-specific analyses indicated attributable fractions of 10.32% (95% eCI: 5.54% to 14.89%) for males and 7.62% (95% eCI: 1.80% to 13.06%) for females. The attributable fraction for the ≥65 years age group was 11.51% (95% eCI: 7.00% to 15.75%), while it was 16.79% (95% eCI: -8.21% to 36.34%) for the 0-64 years age group. Generally, heat was responsible for the majority of the mortality burden, although in the female subgroup, the burdens due to both heat and cold were not statistically significant. Attributable numbers are presented in Table 5.

**Table 4:** The attributable fractions to non-optimal temperatures with 95% empirical confidence interval (95% eCI).

groups	Total (%)	Cold (%)	Heat (%)
Overall	9.21 (5.32 to 12.15)	3.67 (1.45 to 5.80)	5.55 (2.04 to 8.59)
Respiratory diseases	23.80 (15.60 to 30.36)	10.76 (5.38 to 15.72)	13.16 (7.25 to 18.42)
Cardiovascular diseases	11.99 (6.58 to 17.15)	4.16 (1.08 to 6.96)	7.85 (3.10 to 12.09)
male	10.32 (5.54 to 14.89)	4.06 (1.39 to 6.41)	6.28 (2.35 to 10.08)
female	7.62 (1.80 to 13.06)	3.16 (-0.13 to 6.00)	4.48 (-0.94 to 8.88)
≥65 years	11.51 (7.00 to 15.75)	4.63 (1.84 to 7.26)	6.91 (3.29 to 10.39)
0-64 years	16.79 (-8.21 to 36.34)	8.00 (-8.55 to 20.01)	8.92 (-3.69 to 19.40)

**Table 5:** The attributable numbers to non-optimal temperatures with 95% empirical confidence interval (95% eCI).

groups	Total	Cold	Heat
Overall	12902 (6797 to 18101)	5147 (20623 to 7921)	7780 (2818 to 12282)
Respiratory diseases	5004 (3404 to 6544)	2263 (1126 to 3263)	2768 (1473 to 3886)
Cardiovascular diseases	7171 (3762 to 10220)	2488 (739 to 4214)	4696 (1603 to 7532)
male	8593 (4517 to 12237)	3380 (994 to 5280)	5229 (1677 to 8386)
female	4332 (1015 to 7270)	1794 (-140 to 3382)	2546 (-375 to 5298)
≥65 years	11814 (6769 to 16291)	4751 (2046 to 7373)	7093 (3471 to 10593)
0-64 years	6291 (-3438 to 13524)	2997 (-2630 to 7320)	3340 (-1446 to 7239)

In further analyses, we divided temperature into four components: extreme cold (-4.4°C to 0.9°C), mild cold (0.9°C to MMT), mild heat (MMT to 25.8°C), and extreme heat (25.8°C to 28.2°C). As shown in Table 6, in general, mild heat contributed the largest attributable fractions, ranged from 5.18% to 12.42%, while a small fraction of mortality was attributable to extreme cold 0.75% (95% eCI: 0.47% to 1.03%) and extreme heat 0.42% (95% eCI: 0.05% to 0.76%). The female and 0-64 years age subgroups did not show significant attributable risks related to mild cold, mild heat, or extreme heat. Compared to extreme heat,the attributable fraction was higher for extreme cold. Mild heat was responsible for the majority of the mortality burden. Attributable numbers are presented in Table 7.

**Table 6:** The attributable fractions to mild and extreme non-optimal temperature with 95% empirical confidence interval (95% eCI).

groups	Extreme cold (%)	Mild cold (%)	Mild heat (%)	Extreme heat (%)
Overall	0.75 (0.47 to 1.03)	3.01 (0.98 to 4.92)	5.18 (1.74 to 8.24)	0.42 (0.05 to 0.76)
Respiratory diseases	1.11 (0.36 to 1.78)	9.83 (4.54 to 14.47)	12.42 (5.80 to 17.32)	1.11 (0.37 to 1.78)
Cardiovascular diseases	0.87 (0.44 to 1.30)	3.42 (0.72 to 6.03)	7.33 (2.76 to 11.69)	0.64 (0.13 to 1.15)
male	0.81 (0.43 to 1.13)	3.37 (0.88 to 0.57)	5.89 (2.17 to 9.45)	0.44 (-0.04 to 0.87)
female	0.69 (0.24 to 1.09)	2.52 (-0.34 to 5.31)	4.13 (-0.81 to 8.43)	0.40 (-0.14 to 0.91)
≥65 years	0.74 (0.40 to 1.07)	3.99 (1.25 to 6.64)	6.38 (3.00 to 9.60)	0.65 (0.22 to 1.05)
0-64 years	1.21 (0.16 to 2.12)	6.97 (-6.89 to 18.10)	8.79 (-3.26 to 18.01)	0.18 (-0.05 to 0.41)

**Table 7:** The attributable numbers to mild and extreme non-optimal temperature with 95% empirical confidence interval (95% eCI).

groups	Extreme cold	Mild cold	Mild heat	Extreme heat
Overall	1058 (646 to 1442)	4221 (1356 to 7065)	7264 (2639 to 11433)	589 (57 to 1072)
Respiratory diseases	234 (80 to 369)	2067 (890 to 3055)	2612 (1406 to 3641)	233 (60 to 384)
Cardiovascular diseases	523 (275 to 769)	2048 (353 to 3623)	4385 (1458 to 6714)	385 (81 to 691)
male	672 (363 to 942)	2804 (631 to 4842)	4907 (1674 to 7913)	363 (-6 to 705)
female	391 (141 to 618)	1432 (-197 to 3094)	2349 (-459 to 4911)	225 (-85 to 527)
≥65 years	755 (378 to 1088)	4093 (1549 to 6763)	6546 (3173 to 9890)	669 (247 to 1063)
0-64 years	453 (51 to 767)	2610 (-2949 to 6852)	3290 (-1415 to 6750)	68 (-23 to 155)

### Sensitivity analysis

Sensitivity analyses were conducted to examine the robustness of the findings. Specifically, we varied the degrees of freedom (df) for humidity and air pollutants to assess their impact on the estimated temperature-related mortality risks and the minimum mortality temperature. The results, by adjusting the df for humidity across different models, did not significantly alter the temperature-attributable risks, affirming the stability of the findings. As shown in Table 8.

**Table 8:** The impact of different lag days and degrees of freedom (df) for covariates on sensitivity analyses by calculating minimum mortality temperatures (MMTs) and the fraction with 95% empirical confidence interval (95%CI).

Model choices	MMT (°C)	Total (%)	Cold (%)	Heat (%)
**df for humidity**				
2	13.8	9.22 (5.02 to 13.16)	3.69 (1.43 to 5.78)	5.55 (2.03 to 9.02)
4	13.8	9.18 (5.10 to 13.00)	3.66 (1.41 to 5.66)	5.53 (2.01 to 8.51)
5	13.8	9.14 (4.87 to 12.90)	3.64 (1.53 to 5.61)	5.52 (1.87 to 8.77)
**df for SO**_**2**_				
5	13.9	9.25 (5.06 to 13.33)	3.49 (1.52 to 5.69)	5.52 (1.96 to 8.79)
7	13.9	9.23 (5.30 to 13.00)	3.72 (1.47 to 5.83)	5.53 (2.05 to 8.70)
8	13.9	9.20 (5.48 to 13.20)	3.70 (1.26 to 5.72)	5.52 (2.07 to 8.89)
**df for PM**_**2.5**_				
5	13.8	9.19 (4.78 to 12.98)	3.69 (1.50 to 5.75)	5.51 (1.74 to 8.78)
7	13.9	9.22 (5.17 to 13.00)	3.70 (1.55 to 5.81)	5.54 (2.24 to 9.03)
8	13.9	9.21 (5.14 to 12.84)	3.70 (1.70 to 5.62)	5.53 (2.14 to 8.75)

## Discussion

In this study, we applied the DLNM, a time series model, to examine the exposure-response relationships between temperature and non-accidental mortality, stratified by gender, age and cause-specific diseases, over the period from 2013 to 2023. Furthermore, we calculated the attributable fraction and attributable numbers associated with non-optimal temperatures to assess the mortality burden.

The observed U-shaped association between temperature and non-accidental mortality aligns with previous research indicating increased mortality risks at both low and high temperatures [13]. In the various subgroups, except for the respiratory diseases group, the risks associated with low temperature increased as the temperature decreased. This finding is consistent with previous studies [14,15]. Conversely, the risks related to high temperatures initially increased and then decreased as the temperature rose. It is possible that people adopt more proactive strategies to cope with high temperatures, or because methods to mitigate the effects of heat are more readily accessible. And females and individuals aged 0-64 years demonstrated greater adaptability to high temperatures compared to males and older individuals, suggesting that males and older individuals may be more susceptible to heat-related damage. This finding is consistent with the results of other studies [16–18]. One possible explanation is that females generally have a higher proportion of subcutaneous fat, which helps with insulation and maintaining a stable body temperature. Hormonal fluctuations, such as those of estrogen and progesterone, also play a role. Estradiol and progesterone influence thermoregulation both centrally and peripherally, with estradiol promoting heat dissipation and progesterone promoting heat conservation and higher body temperatures, which can further affect temperature regulation [19,20]. Old people exhibit reduced sweating capacity compared to younger individuals, which impairs their ability to regulate body temperature effectively. As a result, increases in heat storage become more pronounced, making it more difficult for their bodies to cool down during periods of elevated temperatures [17].The effects of extreme temperatures revealed that the impact of extreme low temperature generally began 2 to 4 days after exposure and persisted for about 18 days, whereas the effects of extreme high temperature were immediate and shorter in duration, consistent with the results of other studies [21,22]. The impact of extreme heat was not detected in females or individuals aged 0-64 years. Additionally, the temperature-related risks were greater for respiratory diseases compared to cardiovascular diseases. In a heat wave study, the effect estimates for respiratory diseases were higher than those for cardiovascular diseases [23,24]. Furthermore, individuals aged0-64 years were more vulnerable to extreme cold effects. This age group mainly consists of students and workers, therefore they spend more time exposed to outdoor cold air, potentially increasing their susceptibility.

We further assessed the mortality burden by calculating the attributable fractions related to cold and heat effects. Our findings indicated that 9.21% of non-accidental mortality was attributed to non-optimal temperature, which is lower than 11.00% in China reported in global analysis [25] and 11.03% across 17 Chinese cities [26]. Furthermore, by defining the cold and heat effects by mean temperature, we found that the heat effect was responsible for the majority of mortality burden, a finding that diverges the previous studies [25]. Upon separating the temperature into four components, the mortality burden from extreme cold was higher than that from extreme heat, consistent with several reports [27–29]. Given the high risk and prolonged duration associated with extreme cold, this underscores the importance of heightened attention to extreme cold weather events. Several studies support the claim that mild cold was responsible for the largest attributable fraction [9,14,25], however, in our study, mild heat had the largest mortality burden. One possible reason is that Guiyang is located in the subtropical monsoon zone, where the period of mild heat is longer than that of mild cold, thereby leading to a greater attributable burden from mild heat. This comparison highlights the regional particularities in the impact of non-optimal temperatures on mortality rates, emphasizing the necessity of localized recommendations. Moreover, the respiratory mortality burden associated with non-optimal temperatures was 23.80%, representing the largest attributable fraction in this study. A possible reason is that temperature influences pathogen reproduction, and directly exposes the respiratory tract to external air, making the respiratory system more susceptible to temperature changes and airborne infectious diseases. This finding suggests that people with respiratory conditions such as respiratory-transmitted infectious diseases, asthma and chronic obstructive pulmonary disease (COPD), are at greater risk from non-optimal temperatures [30–32]. Although the attributable fraction for cardiovascular mortality was 11.99%, which is lower than that for respiratory mortality, this result aligns with previous studies [33]. Nonetheless, it still highlights the significant need for attention and intervention especially for stroke and coronary heart disease [34,35]. While the mortality burden for individuals aged0-64 years was not statistically significant, males and individuals aged 65 years and above exhibited a higher mortality burden related to non-optimal temperatures.

Several limitations should be noted in this study. First, as an ecological study, our analysis may be subject to ecological fallacy, where population-level associations may not reflect individual temperature-mortality relationships. Second, we did not control for certain socioeconomic factors that may influence the temperature-mortality relationship, such as economic status. Third, although the quality of mortality data was strictly controlled, we cannot exclude the possibility of misdiagnosis or coding errors. These limitations should be considered when interpreting our findings and should be addressed in future research through more detailed individual-level studies and improved data collection methods.

Our findings underscore the need for localized climate adaptation policies in Guiyang, including the development of targeted heat and cold wave response plans. These plans should prioritize vulnerable groups such as the elderly and individuals with pre-existing health conditions. Implementing early warning systems and community outreach programs tailored to these high-risk populations could significantly mitigate the mortality burden associated with temperature extremes.

## Conclusion

The effects of extreme high temperatures had a higher relative risk, occurring immediately, but with a shorter duration compared to the effects of extreme low temperatures. Mild heat contributed the majority of the attributable fraction. Temperature fluctuations were more strongly associated with respiratory diseases. Females and individuals aged 0-64 years exhibited greater adaptability to temperature changes.
